# The influence of hospital accreditation: a longitudinal assessment of organisational culture

**DOI:** 10.1186/s12913-019-4279-7

**Published:** 2019-07-09

**Authors:** Ellie Bostwick Andres, Wei Song, Catherine Mary Schooling, Janice Mary Johnston

**Affiliations:** University of Hong Kong, School of Public Health, Patrick Manson Building, (North Wing), 7 Sassoon Road, Hong Kong, People’s Republic of China

**Keywords:** Accreditation, Organizational theory, Hospital medicin

## Abstract

**Background:**

A growing body of evidence supports the link between hospital organisational culture and health outcomes. Organisational culture is thus an essential consideration for hospital accreditation, a practice of systematically assessing the quality of hospital care against accepted standards. This study assesses the interplay between accreditation and hospital professional staff perception of organisational culture.

**Methods:**

A prospective cohort study design was used to explore the influence of accreditation on organisational culture within a large, publicly-funded, university teaching hospital in Hong Kong. All full-time hospital and academic physicians, nurses and allied health professionals were invited to participate. Organisational culture was evaluated using the Competing Values Framework through the Quality Improvement Implementation Survey. Organisational culture was assessed longitudinally at 9 months prior to accreditation, 3 months following and 15 months after accreditation. To capture potential shifts in staff perception of organisational culture through the accreditation process, we conducted a between time-point comparison using a linear trend model.

**Results:**

545 clinical staff completed the organisational culture survey pre-accreditation, 378 three- months post-accreditation and 141 15-months post-accreditation. Hierarchical culture was the dominant organisational culture domain pre-accreditation, followed by rational, developmental and group culture, respectively. Following accreditation, hierarchical culture declined but remained dominant, while group and developmental culture increased. However, the decline in hierarchical culture was U-shaped with scores increasing at 15-months post-accreditation, though not to pre-accreditation levels. When stratified by professional group, hierarchical culture declined following accreditation with corresponding increases in group culture and developmental culture among physicians and nurses, respectively. While allied health professionals did not perceive any significant cultural differences directly following accreditation, a significant increase in hierarchical culture and corresponding decrease in group culture was found 15-months post-accreditation.

**Conclusions:**

This study suggests the hospital accreditation process may contribute to shifts in staff perception of organisational culture. Our findings also indicate differential views of organisational culture across professional groups. Finally, we note the striking dominance of hierarchical culture in this Hong Kong hospital across all time points, far surpassing other studies, even those in which hierarchical culture prevailed.

**Electronic supplementary material:**

The online version of this article (10.1186/s12913-019-4279-7) contains supplementary material, which is available to authorized users.

## Background

A growing body of evidence supports the link between hospital organisational culture and health outcomes [1]. Organisational culture, a pattern of shared assumptions, values and norms, facilitates a hospital’s approach to coping with complex and dynamic challenges [2, 3]. Health care organisations with cultures characterised by collaboration, flexibility, and risk-taking are most successful implementing quality improvement initiatives, whereas, bureaucratic and hierarchical cultures may inhibit improvement efforts [4–7]. Organisational culture is thus an essential consideration for health care quality improvement initiatives, such as hospital accreditation.

Accreditation is a practice of systematically assessing the quality of hospital care against accepted standards [8]. Successful certification of accreditation signals to patients and other stakeholders that a minimum quality standard has been achieved [9]. This approach to quality improvement is predicated on the expectation that the accreditation exercise leads to improvement in clinical governance and quality of care [10, 11]. While there is limited evidence supporting its effect on patient outcomes, accreditation is nevertheless a thriving industry internationally and is considered an essential quality improvement activity [8, 11, 12].

No prior study has investigated the longitudinal effects of the hospital accreditation process on organisational culture. Likewise, there is no existing indication of hospital organisational culture in Hong Kong, which is likely to differ from western contexts where most studies of organisational culture have been conducted. A robust study of Chinese public hospitals, likely more pertinent to Hong Kong than western hospitals, found employees perceived an organisational culture dominated by internal rules and regulations with limited capacity for capability development, team orientation and empowerment [13].

The Hong Kong public hospital system recently launched a pilot programme to test an infrastructure for accreditation of both private and public hospitals with the Australian Council on Healthcare Standards (ACHS) [14]. This empirical situation offered a rich context for studying the interplay between accreditation and organisational culture. This study aims to assess the longitudinal relationship between accreditation and organisational culture within a Hong Kong public teaching hospital.

## Methods

### Setting and design

This study was part of a prospective mixed methods evaluation of the impact of accreditation on hospital quality, patient experience and organisational culture conducted in a large, publicly-funded, university teaching hospital in Hong Kong. The current paper presents findings from the longitudinal staff assessment of organisational culture. Hospital administrators introduced the first round of the organisational culture survey 9 months prior to the accreditation assessment (T1) as a baseline measurement. Subsequent surveys at 3 months (T2) and 15 months (T3) post-accreditation award measured immediate and enduring effect.

The accreditation intervention began with a gap analysis based on ACHS standards. Between T1 and T2, the hospital undertook a series of quality improvement initiatives to address identified gaps as described in detail elsewhere [14]. The hospital implemented initiatives to address hospital-wide issues, such as improving coordination, reporting and integration as well as specific department and procedure-level gaps.

All full-time hospital and academic clinical staff (*n* = 2781) were invited to participate in the culture survey. Part-time, agency and out-sourced staff who comprise only a small proportion of hospital clinical staff were excluded. To ensure respondent confidentiality, one un-blinded research assistant assigned each staff member included on the staff list provided by the human resources department a randomly generated unique study identifier (USI). All study data were stripped of personal identifiers. The master linking file was kept in a data safe only accessible to the un-blinded research assistant. The un-blinded research assistant assembled envelopes addressed to each participant with their name, rank and department. Envelopes included a cover letter explaining the study and inviting participation, a USI-marked questionnaire and a return envelope addressed to the investigator.

The surveys were sent via internal mail 9 months prior to accreditation (T1). Questionnaires were completed and returned to the research team via internal mail. Subsequent data collection occurred at 3 months (T2) and 15 months (T3) post-accreditation following the same procedure. The un-blinded research assistant crosschecked the USI for returned questionnaires to identify non-responders and send follow-up questionnaires. Two follow-up reminders with questionnaires and return envelopes were sent 2 weeks apart to non-responders. The Institutional Review Board approved this study.

### Study framework and questionnaire

**Fig. 1:**
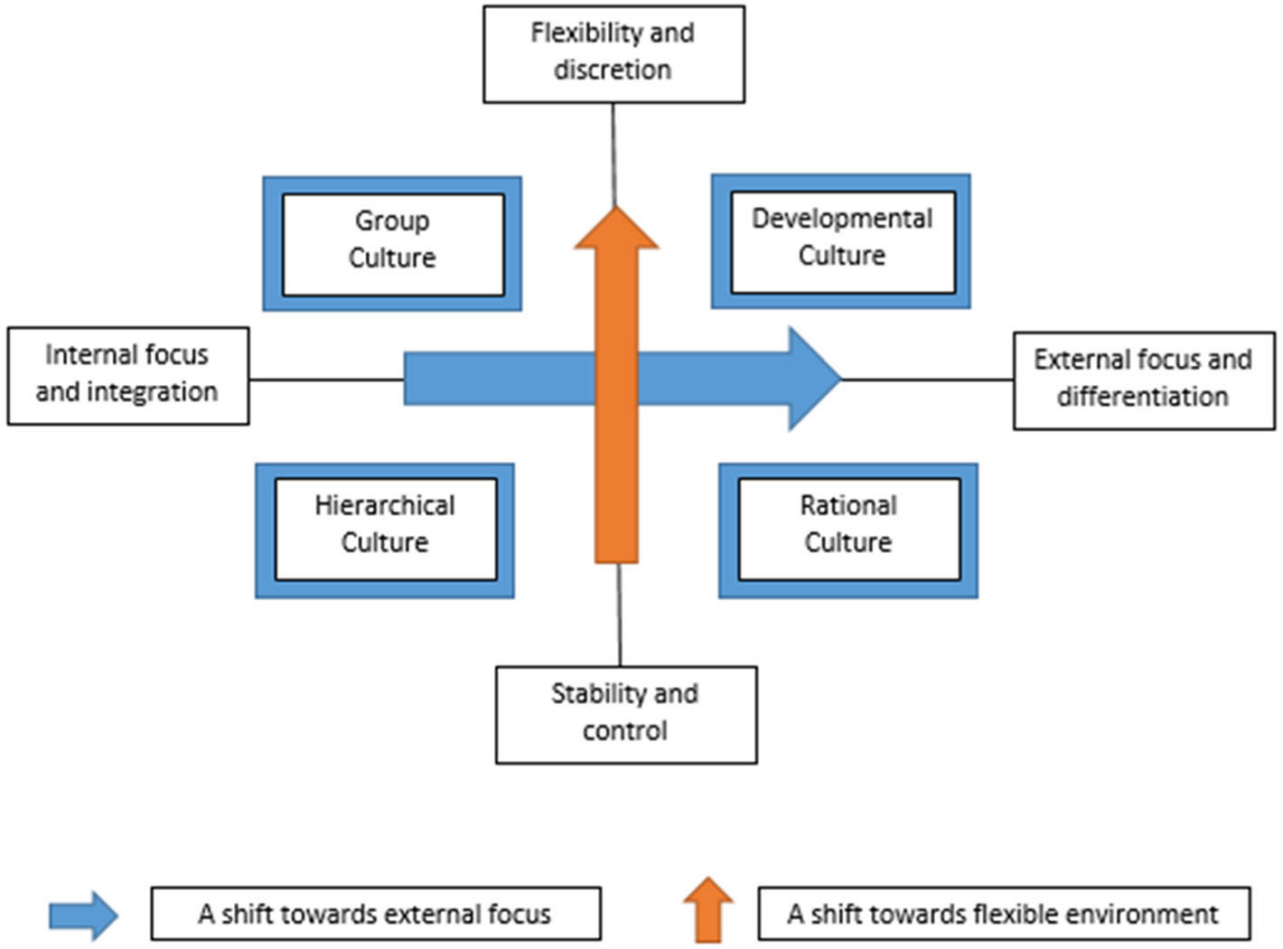
The Competing Values Framework for modeling organizational culture. Adapted from [15]

The concept of organisational culture used in this study is based on the competing values framework (CVF) developed by Quinn and Rohrbaugh [15]. The CVF has been widely used in health services research, including to assess organisational culture as a predictor of quality improvement implementation, employee and patient satisfaction and team functioning [16–19]. The CVF conceptualises organisational culture along two dimensions: structure and focus (Fig. 1). The structural dimension (vertical axis) distinguishes between a culture oriented towards centralisation and control versus decentralisation and flexibility. Likewise, the focus dimension (horizontal axis) ranges from emphasising harmonious internal factors such as employee satisfaction to an external focus on outside entities, including customers and competitors. From these dimensions, organisations can be identified by means of four culture types: group, developmental, rational and hierarchical.

The CVF recognises multiple competing values may coexist within an organisation. It suggests each organisation is comprised of characteristics of the four culture types with varying degrees of emphasis. The CVF also posits that inviting different stakeholder perspectives can enhance quality improvement efforts. The CVF allows for consideration of how the accreditation process contributes to perceived changes in organisational culture by all constituents and to what extent views of culture evolve over time among different professional groups.

With explicit permission from Shortell and colleagues, we selected “The Quality Improvement Implementation Survey (Part 1 Culture)” derived from the CVF to assess perceived changes in organisational culture by hospital employees before and after implementation of hospital accreditation [20]. The questionnaire comprises 20 items divided into five dimensions: Hospital character; Hospital management; Hospital cohesion; Hospital strategic emphases; and Hospital criteria for success. For each dimension, respondents attribute 100 points among the four culture types (group, developmental, rational and hierarchical culture), depending on the extent to which they believe the description matches their hospital. The ipsative scale takes into consideration the interrelated nature of the four quadrants of the CVF and was selected over the Likert scale version to allow for consideration of the holistic nature of organisational culture [21].

Table 1 displays the items for the ‘Hospital character’ dimension and an example of the distribution of points totalling 100. Culture scores represent the mean score for each hospital category (e.g. “Hospital A”), which corresponds with one of the four culture types (e.g. Group culture score = [(Hospital Character Hospital A (Q1) + Hospital’s Managers Hospital A (Q5) + Hospital Cohesion Hospital A (Q9) + Hospital Emphases Hospital A (Q13) + Hospital Rewards Hospital A (Q17)]/n, where n is the number of responses for that set of culture questions). If there were fewer than three valid responses for a culture type, the response was scored missing. Demographic information, including profession, sex, age, education and years in practice was also collected.

**Table 1:** Sample Question Scoring

Question 1 (Q1) Hospital Character (Please distribute 100 points)	
1. *70_Hospital A is a very personal place. It is a lot like an extended family. People seem to share a lot of themselves.*	
2. *30_Hospital B is a very dynamic and entrepreneurial place. People are willing to stick their necks out and take risks.*	
3. *0_* *Hospital C is a very formalized and structured place. Bureaucratic procedures generally govern what people do.*	
4. *0_Hospital D is very production oriented. A major concern is with getting the job done. People aren’t very personally involved.*	

### Data analysis

Descriptive statistics were used to summarize participant demographics. Chi-square and t-test pairwise comparisons evaluated differences in demographic characteristics across time points. While the CVF ipsative scale allows for essential consideration of the interrelated nature of organisational culture, analysis of ipsative data is problematic [21, 22]. Therefore, to capture potential culture shifts pre and post accreditation, we conducted a between time-point comparison (T1 & T2, T1 & T3 and T2 & T3) using a linear trend model. The dependent variable for each model was the mean score in each of the four dimensions of organisational culture (i.e. group, developmental, rational and hierarchical). Time point was incorporated in the model as a covariate. USI was incorporated in the model as a random factor to account for the repeated measure. The model was further stratified by profession to examine group differences over time for doctors, nurses and allied health professionals. Data were analysed using STATA 13. A priori significance level of 0.05 was used for all statistical tests.

## Results

### Participant characteristics

A total of 545 clinical staff (20% of eligible respondents) completed the pre-accreditation survey (T1), including 23% of eligible medical (*N* = 125/577), 18% of eligible nursing (293/1646), and 42% of eligible allied health (127/303) employees. Following accreditation, 378 respondents (69% of T1) completed the questionnaire at T2 and 141 (37% of T2) at T3. There were no significant differences in sociodemographic characteristics between T1 and T2 respondents, except for education, with more highly educated participants at T2. T1 vs T3 respondents differed significantly by profession, education and years in practice. By T3, respondents were more likely to be nurses, have postgraduate degrees and more extensive work experience (Table 2). There were no significant differences in organisational culture scores between participants who dropped out versus those who did not (i.e., T1 vs T2 & T3 responders, T2 vs T3 responders) (See Additional file 1: Table S1)

**Table 2:** Comparison of Participant Characteristics across 3 Time points

Category	T1	T2	T3	Chi-square	Pairwise Comparison *p*-value		
	*n* = 545 (%)	*n* = 378 (%)	*n* = 141 (%)	*p*-value	T1 & T2	T1 & T3	T2 & T3
Gender				0.09	0.42	0.03*	0.12
Male	165 (30.6)	106 (28.1)	30 (21.3)				
Female	374 (69.4)	271 (71.9)	111 (78.7)				
Age Group				0.11	0.27	0.04*	0.36
18–29	93 (17.3)	53 (14.2)	13 (9.2)				
30–39	186 (34.6)	120 (32.1)	43 (30.5)				
40–49	180 (33.5)	132 (35.3)	59 (41.8)				
≥ 50	79 (14.7)	69 (18.4)	26 (18.4)				
Profession				0.00**	0.26	0.00**	0.00**
Medical	125 (22.9)	71 (18.8)	14 (9.9)				
Nursing	293 (53.8)	221 (58.5)	96 (68.1)				
Allied Health	127 (23.3)	86 (22.8)	31 (22.0)				
Education				0.00**	0.04*	0.00**	0.16
Vocational	49 (9.2)	23 (6.1)	5 (3.5)				
Undergrad	232 (43.4)	144 (38.5)	52 (36.9)				
Postgraduate	253 (47.4)	207 (55.3)	84 (59.6)				
Years in Practice				0.07	0.15	0.04*	0.34
≤ 10	176 (32.3)	103 (27.3)	30 (21.3)				
> 10–20	211 (38.7)	146 (38.6)	62 (44.0)				
> 20	158 (29.0)	129 (34.1)	49 (34.8)				

*p < .05, **p < .01

### Organisational culture

Hierarchical culture was the dominant organisational culture domain at T1 (pre-accreditation), comprising 42 of 100 points, followed by rational (27 points), developmental (16 points), and group (15 points) culture (Table 3). After accreditation (at T2), the largest absolute change in the domain mean culture scores was for the hierarchical culture score which declined (T1 vs T2 = -2.39 points; *p* for trend <.01). However, this decline was U-shaped with the hierarchical culture score increasing from T2 to T3, though not to pre-accreditation levels. Corresponding with the declining hierarchical scores at T2, group and developmental culture domain scores increased following accreditation and these increases were maintained at T3 (Table 4). The individual items contributing most to the domain score shifts included the declining scores for ‘rule enforcers’ and ‘glued by formal rules and policies’ within the hierarchical domain and increasing scores for ‘glued by loyalty and tradition’ in the group culture and ‘dynamic and entrepreneurial place’ and ‘risk takers’ in the developmental culture domains. The rational culture domain saw the least change in the overall domain and individual item mean scores across all time points.

**Table 3:** Trend analysis of changes in organizational culture for all participants

Dominant culture	Time point				Between time-point comparison (Trend)	
	T1 (N = 545)	T2 (N = 378)	T3 (N = 141)	1 & 2	1 & 3	2 & 3
	Mean (95%CI)	Mean (95%CI)	Mean (95%)	Coef. (P > |Z|)		
Group Culture	**14.92 (13.90–15.95)**	**16.45 (15.23–17.68)**	**16.24 (14.50–17.97)**	1.44 (0.02)	0.74 (0.10)	−0.57 (0.52)
*Personal place*	12.83 (11.30–14.36)	14.12 (12.29–15.95)	11.80 (9.35–14.25)			
*Warm and caring*	15.39 (13.92–16.85)	17.09 (15.36–18.83)	17.91 (15.02–20.80)			
*Glued by loyalty and tradition*	18.31 (16.77–19.85)	20.87 (18.95–22.78)	19.96 (16.55–23.36)			
*Emphasize human resource*	15.12 (13.80–16.44)	15.69 (14.00–17.38)	16.89 (13.66–20.11)			
*Rewards fairly equally*	13.07 (11.58–14.56)	14.62 (12.75–16.48)	14.64 (11.74–17.53)			
Developmental Culture	**16.07 (15.18–16.95)**	**16.84 (15.80–17.88)**	**16.69 (14.98–18.41)**	1.00 (0.05)	0.42 (0.33)	−0.22 (0.81)
*Dynamics and entrepreneurial place*	13.56 (12.23–14.89)	16.30 (14.57–18.03)	14.1 (11.43–16.6)			
*Risk takers*	10.50 (9.30–11.70)	12.11 (10.62–13.60)	10.99 (8.39–13.58)			
*Glued by commitment to innovation and development*	16.82 (15.47–18.17)	17.73 (16.05–19.41)	18.09 (15.11–21.06)			
*Emphasize growth and new resources*	20.72 (19.23–22.21)	19.18 (17.60–20.76)	20.54 (17.72–23.36)			
*Rewards based on individual initiative*	18.84 (17.33–20.36)	18.67 (17.07–20.28)	19.84 (16.64–23.04)			
Hierarchical Culture	**41.88 (40.33–43.43)**	**39.47 (37.74–41.19)**	**40.76 (37.86–43.67)**	−2.67 (< 0.01)	−0.74 (0.29)	1.83 (0.15)
*Formalized and structured place*	44.69 (42.65–46.72)	42.80 (40.35–45.25)	47.09 (42.93–51.26)			
*Rule enforcers*	49.20 (46.85–51.56)	44.69 (42.02–47.37)	46.32 (41.78–50.86)			
*Glued by formal rules and policies*	38.86 (36.81–40.91)	35.27 (33.01–37.52)	37.43 (33.68–41.19)			
*Emphasize permanence and stability*	35.19 (33.18–37.20)	34.17 (31.81–36.52)	34.02 (30.00–38.04)			
*Rewards based on rank*	41.37 (38.69–44.05)	40.28 (37.25–43.31)	38.94 (33.49–44.40)			
Rational Culture	**27.13 (26.23–28.03)**	**27.24 (26.12–28.36)**	**26.31 (24.59–28.02)**	0.18 (0.77)	− 0.44 (0.29)	−0.99 (0.29)
*Production oriented*	28.92 (27.12–30.72)	26.78 (24.71–28.85)	27.09 (23.84–30.34)			
*Coordinators and coaches*	24.91 (23.39–26.43)	26.10 (24.30–27.91)	24.79 (22.06–27.51)			
*Glued by tasks and goal accomplishment*	26.01 (24.51–27.51)	26.14 (24.36–27.92)	24.52 (21.58–27.47)			
*Emphasize competitive actions and achievement*	28.97 (27.19–30.74)	30.96 (28.86–33.06)	28.55 (24.76–32.34)			
*Rewards based on achievement of objectives*	26.72 (25.01–28.43)	26.42 (24.45–28.40)	26.57 (23.13–30.02)

**Table 4:** Trend analysis of changes in organizational culture by three clinical professional groups

Category	Mean Score by Time Point			Between time-point comparison (Trend)		
	1	2	3	1 & 2	1 & 3	2 & 3
				Coef. (P > |Z|)		
**Doctors**	(N = 125)	(*N* = 71)	(N = 14)			
Group Culture	13.40	15.64	13.71	2.01 (0.15)	−0.20 (0.88)	−1.58 (0.38)
Developmental Culture	15.26	15.84	15.91	.62 (0.59)	0.65 (0.63)	−0.49 (0.85)
Hierarchical Culture	43.46	39.93	43.16	−3.86 (0.04)*	−0.91 (0.69)	3.18 (0.43)
Rational Culture	27.87	28.59	27.21	1.15 (0.42)	0.36 (0.78)	−1.04 (0.72)
**Nurses**	(*N* = 293)	(*N* = 221)	(*N* = 96)			
Group Culture	15.67	16.42	17.77	0.77 (0.35)	1.08 (0.06)	0.83 (0.47)
Developmental Culture	16.12	17.53	18.02	1.56 (0.02)*	1.00 (0.07)	0.47 (0.68)
Hierarchical Culture	41.22	38.57	38.21	−2.76 (0.02)*	−1.49 (0.08)	0.37 (0.82)
Rational Culture	26.99	27.48	26.00	0.46 (0.59)	−0.60 (0.25)	−1.52 (0.20)
**Allied health**	(*N* = 127)	(*N* = 86)	(*N* = 31)			
Group Culture	14.71	17.21	12.63	2.45 (0.07)	0.11 (0.88)	−4.01 (0.02)*
Developmental Culture	16.73	15.91	12.95	−0.18 (0.87)	−1.46 (0.04)*	−2.72 (0.10)
Hierarchical Culture	41.85	41.34	47.59	−1.26 (0.49)	2.22 (0.15)	5.24 (0.04)*
Rational Culture	26.71	25.54	26.84	−1.05 (0.37)	−0.03 (0.97)	1.36 (0.45)

*p < .05, **p < .01

### Organisational culture and professional group.

When stratified by professional group, doctors and nurses indicate a significant decline in hierarchical culture following accreditation in line with overall trends (Table 4). However, doctors’ hierarchical scores rebound to near pre-accreditation levels at T3, whereas nurses’ perceived decline is sustained. As hierarchical scores decline and rebound for doctors, group culture scores correspondingly increase at T2 and decline at T3, whereas nurses indicate a sustained corresponding increase in developmental culture. Allied health professionals do not perceive any significant cultural differences directly following accreditation at T2, however, they indicate a significant increase in hierarchical culture at T3 and a corresponding decrease in group culture.

## Discussion

This study is the first to evaluate the longitudinal effects of hospital accreditation on organisational culture. Our findings suggest the hospital accreditation process may contribute to shifts in staff perception of organisational culture. In our study, hierarchical culture declined in prominence following accreditation while group culture increased. This shift towards greater flexibility could reflect the influence of the accreditation process, which requires structural and procedural changes to facilitate quality, safety and performance improvement [11, 12, 23] However, despite the initial shifts in organisational culture observed in our study, accreditation’s potential for sustainably transforming organisational culture at the hospital level was not supported by our final time point results. While there was a modest cultural shift away from control towards flexibility between pre-accreditation (T1) and 15-months post-accreditation (T3), the trend was not statistically significant at the aggregate level.

The transient nature of the culture change observed in our study could be attributed to employees’ initial optimism for the accreditation process leading to cultural change through adoption of novel administrative procedures and quality practices [24]. Yet, as employees perceived changes associated with accreditation might not be sustained or encourage the cultural shift anticipated, their hopeful views likewise dissipated. Health care organisations have unique cultural orientations that are difficult to modify [25, 26]. The presence of multiple subcultures fully formed and embedded in routines and organisational structures with clear occupational boundaries are often cited as key impediments to successful organisational culture change [25].

Our study also indicates differential views of organisational culture across professional groups consistent with prior studies [27, 28]. For doctors, a significant decline in hierarchical culture coincided with a rise in group culture, which gradually weakened. This finding is consistent with a recent study in Qatar, which found a positive association between group culture and accreditation in primary care organisations [29]. Moreover, it suggests a potentially valuable contribution of the accreditation process, as research has demonstrated a positive relationship between group-oriented culture and perceived better outcomes of care and clinical efficiency [30]. For nurses, the decline in hierarchical culture coincided with an increase in developmental culture. This shift could reflect changes related to the accreditation process, which has been shown to empower nurses through decentralised decision-making and active involvement in improving care [31, 32]. This theory was supported by findings from our qualitative interviews with nurses at 3-months post-accreditation (to be published), which suggested a major shift in both culture and practice, emphasising autonomy in operational decision-making, despite an increased workload related to accreditation.

Allied health professionals differed from the other professional groups with no significant organisational culture changes observed immediately following accreditation and a significant shift away from group culture towards hierarchical culture between T2 and T3. There was also a modest decline in developmental culture over the study period from T1 to T3. These findings correspond with a prior study that found allied health professionals perceived the accreditation exercise as highly bureaucratic, inefficient and expensive [33]. This sense was also echoed in our interviews with allied health staff, who indicated they felt marginalised and left out of the changes associated with accreditation.

Lastly, while not the primary aim of this study, we note the striking dominance of hierarchical culture in the study hospital across all time points. As a public institution, hierarchy is an expected cultural norm, however, the dominance of hierarchical culture in the study hospital far surpassed other studies, even those in which hierarchical culture dominated [4, 18, 27, 28, 34, 35]. Conversely, group culture, characterised by belonging, trust and participation, is less prevalent than in prior literature. These findings may indicate the pervasive influence of Chinese culture in Hong Kong, which emphasises the importance of strict adherence to hierarchy and prescribed social roles [36]. Our findings support earlier work indicating Chinese hospitals tend to focus on stability and order rather than team orientation and empowerment [37]. They also demonstrate how the values of the society in which an organisation is nested affect organisational values [2–38].

This study is subject to limitations. First, this study was exploratory research aiming to assess the impact of hospital accreditation on changes in organisational culture for one hospital. The single case study used here may not be generalised to other hospitals. Second, the relatively small sample size in the follow-up surveys as a result of low response rates could indicate attrition bias through selective drop out of respondents. Participants who were lost to follow-up were less educated with less experience. As such, these variables could have affected the extent and dimension of the perceived changes in organisational culture. Third, organisational culture was measured by self-report, which could allow for response bias in assessing perceived values or subjective judgements. A rigorous multi-method approach including systematic observation, in-depth interview and archival data analysis could enrich understanding of organisational culture and culture changes [39]. Finally, the nature of our data using an ipsative scale limited our analysis options, as many of the standard statistical methods do not yield valid results [22]. Thus, our analysis focused on the longitudinal changes in prominence of organisational cultures.

## Conclusion

This study offers important insights into the longitudinal shifts in organisational culture experienced by a Hong Kong public hospital during the hospital accreditation process. Overall, we documented positive shifts toward group and developmental culture and away from hierarchical culture through implementation of accreditation standards. However, the encouraging cultural shifts were not enduring at the aggregate level beyond the accreditation process. Sustained efforts to maintain culture change are necessary for lasting impact. Recent awareness of the relationship between organisational culture and health care quality has inspired some to invest in quality improvement initiatives in which fostering organisational culture change is the primary intervention [3, 40]. Such initiatives utilise approaches based on the diffusion of innovation theory to promote organisational cultures associated with high-performing hospitals, with the ultimate goal of improving patient outcomes [41]. Our findings can provide helpful insights into influencing hospital organisational culture to promote hospital quality and performance. Likewise, we note the differential effect of accreditation by professional group. Our study underscores the importance of adopting a multidimensional approach in effecting culture change to embrace competing and divergent professional views.

## Additional file

Additional file 1: Comparison of Organisational Culture Scores among Participants versus Dropouts by Timepoint. (DOCX 18 kb)

## Data Availability

The (de-identified) datasets generated and analysed during the current study are available from the corresponding author on reasonable request.

## Abbreviations

ACHS: Australian Council on Healthcare Standards
CVF: Competing Values Framework
T1, T2, T3: Time point 1 (Baseline: 9 months pre-accreditation), Time point 2 (3 months following accreditation) & Time point 3 (15 months following accreditation)
USI: Unique study identifier
