# Supplementary material for: The influence of hospital accreditation: a longitudinal assessment of organisational culture
Source: BMC Health Serv Res. 2019 Jul 9;19:467. doi: 10.1186/s12913-019-4279-7 (PMC6617556; doi:10.1186/s12913-019-4279-7)
Supplement: Supplementary file 1 — Comparison of Organisational Culture Scores among Participants versus Dropouts by Timepoint. (DOCX 18 kb) [file 12913_2019_4279_MOESM1_ESM.docx]

Additional fie :1 Table S1. Comparison of Participants versus Dropouts (Drops) by Time point

| **Time point** |  |  | **T1** |  |  |  |  | **T2** |  |
| --- | --- | --- | --- | --- | --- | --- | --- | --- | --- |
| **Respondents** | **All (n=545)** | **T2 Drops (n=168)** | **T3 Drops (n=236)** | **T3 (n=141)** | **T2 Drops v. T2 & T3** | **T3 Drops v. T3 Resp** | **T3 Drops (n=236)** | **T3 (n=141)** | **T3 Drops v. T3** |
| **Culture Score** | **Mean (95% CI)** | | | | **p-value** |  | **Mean (95% CI)** | | **p-value** |
| Group | 14.92  (13.90 - 15.95) | 14.47  (12.70-16.23) | 15.59  (14.00-17.18) | 14.35  (12.25-16.45) | 0.56 | 0.35 | 16.11  (14.59-17.62) | 17.04  (14.96-19.11) | 0.47 |
| Developmental | 16.07  (15.18- 16.95) | 16.98  (15.24-18.72) | 15.77  (14.49-17.07) | 15.44  (13.79-17.09) | 0.17 | 0.75 | 16.80  (15.42-18.18) | 16.91  (15.33-18.48) | 0.92 |
| Hierarchical | 41.88  (40.33 - 43.43) | 40.85  (37.87-43.83) | 42.05  (39.76-44.33) | 42.85  (39.85-45.86) | 0.38 | 0.67 | 40.02  (37.89-42.14) | 38.55  (35.57-41.52) | 0.42 |
| Rational | 27.13 (26.23 - 28.03) | 27.71  (25.97-29.45) | 26.58 (25.27-27.90) | 27.35  (25.63-29.07) | 0.39 | 0.48 | 27.08  (25.62-28.55) | 27.51  (25.75-29.26) | 0.72 |
| **Demographics** |  |  |  |  |  |  |  |  |  |
| **Gender** | **n (%)** |  |  |  | 0.06 | 0.02* |  |  |  |
| Male | 165 (30.6) | 60 (36.1) | 75 (32.3) | 29 (21.0) |  |  |  |  |  |
| Female | 374 (69.4) | 106 (63.9) | 159 (67.7) | 109 (79.0) |  |  |  |  |  |
| **Age** |  |  |  |  | 0.69 | 0.46 |  |  |  |
| 18-29 | 93 (17.3) | 28 (17.0) | 46 (19.7) | 19 (13.7) |  |  |  |  |  |
| 30-39 | 186 (34.6) | 64 (38.2) | 73 (31.2) | 50 (36.0) |  |  |  |  |  |
| 40-49 | 180 (33.5) | 51 (30.9) | 79 (33.8) | 50 (36.0) |  |  |  |  |  |
| ≥50 | 79 (14.7) | 23 (13.9) | 36 (15.4) | 20 (14.4) |  |  |  |  |  |
| **Profession** |  |  |  |  | 0.00** | 0.00** |  |  |  |
| Medical | 125 (22.9) | 54 (32.1) | 57 (24.2) | 14 (9.9) |  |  |  |  |  |
| Nursing | 293 (53.8) | 73 (43.5) | 124 (52.5) | 96 (68.1) |  |  |  |  |  |
| Allied Health | 127 (23.3) | 41 (24.4) | 55 (23.3) | 31 (22.0) |  |  |  |  |  |
| **Education** |  |  |  |  | 0.00** | 0.90 |  |  |  |
| Vocational | 49 (9.2) | 25 (15.4) | 16 (6.8) | 8 (5.8) |  |  |  |  |  |
| Undergrad | 232 (43.4) | 75 (46.3) | 100 (42.6) | 57 (41.6) |  |  |  |  |  |
| Postgraduate | 253 (47.4) | 62 (38.3) | 119 (50.6) | 72 (52.6) |  |  |  |  |  |
| **Practice years** |  |  |  |  | 0.48 | 0.66 |  |  |  |
| ≤10 | 176 (32.3) | 60 (35.7) | 74 (31.4) | 42 (29.8) |  |  |  |  |  |
| >10-20 | 211 (38.7) | 60 (35.7) | 97 (41.1) | 54 (38.3) |  |  |  |  |  |
| >20 | 158 (29.0) | 48 (28.6) | 65 (27.5) | 45 (31.9) |  |  |  |  |  |
